# GLOBAL CLINICIAN PERSPECTIVES ON NON-OPERATIVE HIP FRACTURE MANAGEMENT DURING COVID-19

**DOI:** 10.1093/geroni/igac059.3126

**Published:** 2022-12-20

**Authors:** Lucille Xiang, Mriganka Singh, Lynn McNicoll, Iain Moppett

**Affiliations:** Brown University, Providence, Rhode Island, United States; Brown University, Providence, Rhode Island, United States; Brown University, Providence, Rhode Island, United States; University of Nottingham, Nottingham, England, United Kingdom

## Abstract

There are currently no guidelines regarding clinician decision making in the type of hip fracture management among older adults. Cultural, social, structural and economic differences between global healthcare systems may result in differing approaches. This study’s objectives were to identify possible factors influencing clinicians’ decision to undertake a non-operative hip fracture management approach among older adults, and to determine whether there is global heterogeneity regarding these factors between high income countries (HIC), and low- and middle-income countries (LMIC) clinicians. A SurveyMonkey questionnaire was distributed to clinicians through the Fragility Fracture Network’s Perioperative Special Interest Group and clinicians’ personal networks between May 24 and July 25, 2021. 406 respondents from 51 countries returned the questionnaire, of which 225 respondents came from HIC and 180 from LMIC. Clinicians from HIC reported a greater median [IQR] estimated proportion of admitted patients with hip fracture undergoing surgery (96% [95–99]) than those from LMIC (85% [75–95]) of mean (SD) at 94% (8) compared to 81% (16) among LMIC clinicians (p=2.94e-23). Several factors seemed to influence the clinician hip fracture management decision making process. Global heterogeneity seems to exist between HIC and LMIC clinicians regarding factors such as anticipated life expectancy, ability to pay, treatment costs, insufficient resources, and perception of risk in hip fracture management decision-making. This is the first international sampling of clinician perspectives regarding hip fracture management. Further research is necessary for the development of best practice guidelines to improve hip fracture management decision-making and quality of hip fracture care among older adults.

